# Metabolic modeling predicts perturbations extending lifespan in yeast and counteracting aging in mammalian muscle

**DOI:** 10.1186/1753-6561-6-S3-P54

**Published:** 2012-06-01

**Authors:** Keren Yizhak, Orshay Gabay, Haim Cohen, Eytan Ruppin

**Affiliations:** 1 The Blavatnik School of Computer Science, Tel Aviv University, Tel Aviv 69978, Israel; 2 The Mina and Everard Goodman Faculty of Life Sciences, Bar-llan University, Ramat-Gan 52900, lsrael; 3 The Sackler School of Medicine, Tel Aviv University, Tel Aviv 69978, Israel

## Background

Disease is classically viewed as a disruption of healthy homeostasis. This naturally gives rise to the quest to find drugs that can efficiently transform a disease state back to a healthy one. Here we address this challenge on a genome scale for the first time. We chose to focus on aging, aiming to predict perturbations (both genetic and environmental) that can extend the organism’s lifespan. Aging forms a nice test bed to examine our approach, since it is typically accompanied by progressive changes in gene expression. Furthermore, Caloric Restriction (CR), a dietary intervention that extends lifespan and delays the onset of age-associated phenotypes, is known to reverse these expression changes [1,2]. As CR has very limited value as a therapeutic regimen, these findings strongly motivate the search for metabolic drug targets that can reverse the metabolic state of the aging to that of the young.

## Materials and methods

Here we present a novel Metabolic Transformation Algorithm (MTA) that given a source (disease, e.g., aged) and a desired target (healthy, e.g., young) metabolic state, identifies the genetic or environmental perturbations that best enable a transformation from the source to the target state. The MTA algorithm works in the realm of metabolism and is based on Constraint-Based Modeling, an increasingly widely used computational method for studying metabolism on a genome-scale [3,4].

## Results

First, the prediction accuracy of MTA has been extensively validated using data from known perturbations in *Escherichia coli*, *Saccharomyces cerevisiae* and mammalian cell lines. Second, Analyzing gene expression data in aging *Saccharomyces cerevisiae*, seven novel lifespan-extending metabolic targets predicted by MTA were further tested experimentally. Two of those were successfully validated (a 10-fold increase over their expected frequency), one of them extending lifespan markedly by about 50%. Analyzing mammalian aging muscle expression data, MTA identifies novel drug targets transforming the metabolic state to that of the young, highlighting the role of a key inflammatory pathway of Eicosanoids metabolism. These predictions are enriched with human orthologs of known lifespan-extending genes in *Saccharomyces cerevisiae* and *Caenorhabditis elegans.*

## Conclusions

MTA offers a fundamentally new approach for identifying metabolic drug targets in a broad span of major metabolically-related human disorders, including obesity, neurodegeneration and cancer. As MTA aims to retrieve the metabolic state back to its normal homeostasis, one may expect that it may lead to new drugs with lesser side-effects.
